# Prognostic value of the Geriatric Nutritional Risk Index in sepsis-associated acute kidney injury: a retrospective cohort study

**DOI:** 10.3389/fnut.2025.1635568

**Published:** 2025-11-21

**Authors:** Guangqin Ren, Huali Tang, Xue Guo, Yunxue He, Zhengrong Ding, Zhiwei Li, Cong Zhou, Bin Li, Huibing Chen, Lijuan Dong

**Affiliations:** 1 The Tenth Clinical Medical College of Guangzhou University of Traditional Chinese Medicine, Zhongshan, China; 2 Zhongshan Hospital of Traditional Chinese Medicine Affiliated to Guangzhou University of Traditional Chinese Medicine, Zhongshan, China; 3 Zhongshan Hospital of Traditional Chinese Medicine, Zhongshan, China; 4 Information Engineering University of the People’s Liberation Army Cyberspace Force, Zhengzhou, China

**Keywords:** Geriatric Nutritional Risk Index, sepsis-associated acute kidney injury, continuous renal replacement therapy, short-term mortality, nutritional assessment

## Abstract

**Background:**

The Geriatric Nutritional Risk Index (GNRI) is a simple and objective tool for assessing nutritional status. It has shown prognostic value in patients with acute kidney injury (AKI). However, evidence in sepsis-associated AKI (S-AKI) remains limited, especially among patients receiving continuous renal replacement therapy (CRRT).

**Methods:**

This retrospective cohort study included 773 critically ill S-AKI patients who received CRRT, using data from the publicly available Dryad database. Patients were stratified into tertiles based on GNRI values. Cox proportional hazards models were employed to assess the association between GNRI and 28-day and 90-day all-cause mortality, with the lowest tertile serving as the reference group. Kaplan–Meier survival analyses were used to compare cumulative mortality across GNRI strata.

**Results:**

Among 773 patients, the 28-day and 90-day mortality rates were 61.7% and 73.0%, respectively. After adjusting for multiple confounders, higher GNRI scores were independently associated with lower all-cause mortality. Compared to the lowest GNRI group, the highest tertile showed significantly reduced mortality risks (28-day HR = 0.53; 95% CI: 0.47–0.75; *p* < 0.001; 90-day HR = 0.50; 95% CI: 0.41–0.63; *p* < 0.001). The association remained robust in subgroup analyses and was particularly pronounced in patients aged ≥65 years.

**Conclusion:**

GNRI independently predicts short-term mortality in critically ill S-AKI patients on CRRT. It is simple, objective, and integrates nutritional and inflammatory status. It can assist early risk stratification and nutritional assessment.

## 1 Introduction

Sepsis is a severe and systemic response to infection that frequently progresses to multi-organ dysfunction. Acute kidney injury (AKI) is among the most prevalent and devastating complications of sepsis, leading to significantly higher mortality and worse clinical outcomes compared to non-septic AKI cases (1, 2). This specific phenotype, referred to as sepsis-associated acute kidney injury (S-AKI), accounts for up to 40–60% of AKI cases in intensive care units (ICUs) and presents unique diagnostic and therapeutic challenges (3). Despite continuous advances in renal replacement therapies, including continuous renal replacement therapy (CRRT), mortality among patients with severe S-AKI remains alarmingly high, often exceeding 50% (4, 5).

Malnutrition is increasingly recognized as a key modifiable factor contributing to adverse outcomes in critically ill patients with AKI (6). Accurate assessment of nutritional status in this population, however, remains challenging. Conventional methods such as the Subjective Global Assessment (SGA), body mass index (BMI), and anthropometric measurements are widely used to assess nutritional status. However, in ICU patients, these methods are often unreliable because of fluid shifts, electrolyte imbalances, and hemodynamic instability (7). These limitations are particularly prominent in patients with S-AKI, in whom aggressive fluid resuscitation may mask underlying malnutrition.

The Geriatric Nutritional Risk Index (GNRI) is calculated from serum albumin levels and the ratio of actual to ideal body weight. It provides a simplified and objective measure of nutritional risk (8). GNRI has also been validated as a prognostic marker in several population, including patients with chronic kidney disease (9), acute heart failure (10) and those undergoing cardiovascular (11). Recent studies have shown that higher GNRI scores predict better short-term outcomes in patients with AKI (12, 13). However, evidence on its prognostic role in S-AKI patients receiving CRRT remains limited.

The study aimed to examine the association between GNRI and short-term mortality in the critically ill patients with S-AKI undergoing CRRT. We hypothesized that GNRI, as a practical and readily available nutritional indicator, would serve as an independent predictor of 28-day and 90-day all-cause mortality in this high-risk population. Our findings may provide a basis for incorporating nutritional risk assessment into early management strategies for patients with S-AKI.

## 2 Materials and methods

### 2.1 Study design and data source

This study is a secondary analysis of a retrospective cohort published by Jung et al. (14), available in the Dryad Digital Repository (DOI: 10.1371/journal.pone.0191290). The dataset comprised critically ill patients with severe AKI who received CRRT at two tertiary hospitals in South Korea, namely Severance Hospital of Yonsei University and the National Health Insurance Service Ilsan Hospital, between 2009 and 2016.

This study analyzed de-identified data from a publicly available cohort ( doi: 10.1371/journal.pone.0191290). Ethical approval was obtained from Yonsei University Severance Hospital IRB (No. 4-2016-1073) with waived informed consent, adhering to the Declaration of Helsinki.

### 2.2 Study population

**FIGURE 1:**
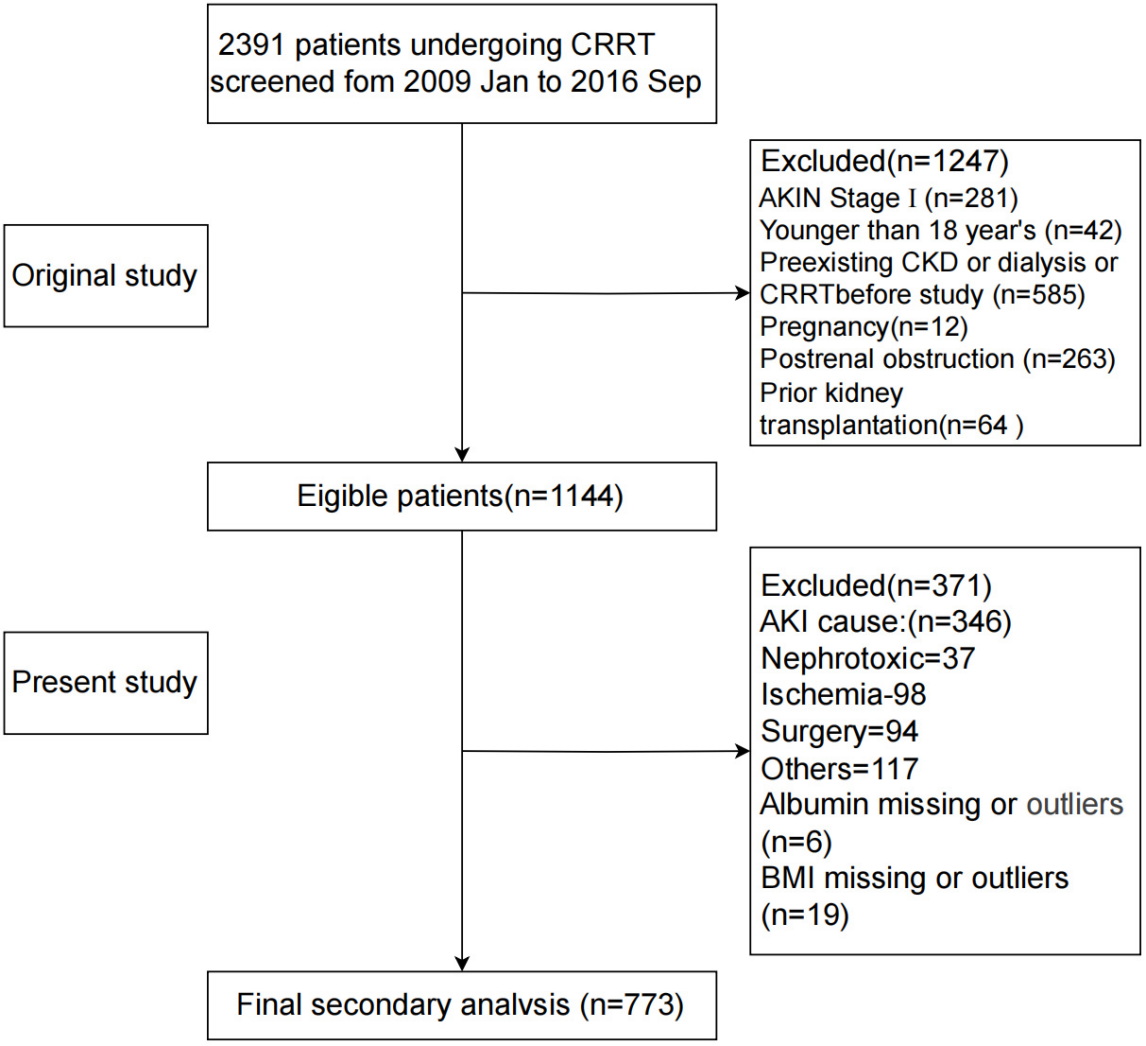
Flowchart of participant screening. AKIN, Acute Kidney Injury Network; AKI, acute kidney injury; BMI, body mass index; CKD, chronic kidney disease; CRRT, continuous renal replacement therapy; GNRI, Geriatric Nutritional Risk Index.

A total of 2,391 patients undergoing CRRT were initially screened. Inclusion criteria were based on the Acute Kidney Injury Network (AKIN) classification, with only stage 2 or 3 AKI patients included (15). Exclusion criteria were: AKI stage 1 (*n* = 281), Age <18 years (*n* = 42), Chronic kidney disease or dialysis dependency (*n* = 585), Pregnancy (*n* = 12), Post-renal obstruction (*n* = 263), Renal transplant recipients (*n* = 64). In addition, patients with non-sepsis-related AKI (e.g., nephrotoxic, ischemic, or postoperative etiologies) and those with missing or invalid data for serum albumin or BMI were. The final cohort included 773 patients diagnosed with S-AKI who underwent CRRT (Figure 1).

### 2.3 Exposure and outcomes

The primary exposure variable was the GNRI, a validated screening tool for assessing nutritional status in hospitalized patients (16). GNRI was calculated before the initiation of CRRT using the following formula: GNRI = (14.89 × serum albumin in g/dL) + (41.7 × actual weight/ideal weight). Ideal body weight was determined based on height and a standard body mass index (BMI) of 22 kg/m^2^. For patients whose actual weight exceeded their ideal weight, the weight ratio was set to 1. GNRI was analyzed both as a continuous variable and as a categorical variable. In this study, GNRI was divided into tertiles based on its distribution and in reference to previous literature (13, 17). The tertiles were defined as follows: T1: GNRI <77.3, T2: GNRI 77.3–87.2, and T3: GNRI ≥87.2.

The primary outcomes were 28-day and 90-day all-cause mortality following the initiation of CRRT. Survival status was obtained from the original dataset.

### 2.4 Clinical and laboratory variables

Baseline data were collected at the time of CRRT initiation and included demographic information (age, sex, BMI), comorbidities [myocardial infarction, congestive heart failure, peripheral vascular disease, dementia, diabetes mellitus (DM), hypertension, chronic obstructive pulmonary disease (COPD)], and vital signs. Laboratory indices included serum albumin (ALB), hemoglobin, phosphate, creatinine (Cr), blood urea nitrogen (BUN), C-reactive protein (CRP), potassium, and estimated glomerular filtration rate (eGFR). Disease severity was assessed using the Acute Physiology and Chronic Health Evaluation II (APACHE II) (18) and Sequential Organ Failure Assessment (SOFA) scores (19).

### 2.5 Statistical analysis

Continuous variables with normal distributions were presented as mean ± standard deviation (SD), while non-normally distributed variables were expressed as median (interquartile range, IQR). Categorical variables were summarized as frequencies and percentages. Group comparisons were performed using one-way ANOVA or Kruskal-Wallis tests for continuous variables, depending on data distribution, and chi-square or Fisher’s exact tests for categorical variables, as appropriate. To minimize bias from missing data, variables with more than 10% missingness were excluded, while those with less than 10% missingness were imputed using multiple imputation. We used Kaplan–Meier curves and log-rank tests to compare 28-day and 90-day survival across GNRI tertiles. Cox proportional hazards regression models were used to evaluate the association between GNRI and mortality. Results were reported as hazard ratios (HRs) with 95% confidence intervals (CIs). The proportional hazards (PH) assumption was tested using Schoenfeld residuals. Potential covariates were selected based on their clinical relevance and evidence from previous studies (17, 20). Four sequential models were constructed. The crude model was unadjusted. Model I was adjusted for age and sex. Model II was further adjusted for mean arterial pressure and comorbidities, including myocardial infarction, congestive heart failure, peripheral vascular disease, dementia, diabetes mellitus, hypertension, and chronic obstructive pulmonary disease. Model III additionally incorporated laboratory parameters (phosphate, hemoglobin, and C-reactive protein), disease severity scores (APACHE II and SOFA), and AKIN stage. Subgroup analyses were performed to assess the robustness of the findings, stratifying patients by age (< 65 vs ≥ 65 years), sex (Male vs Female), DM (No vs Yes), SOFA (< 12 vs ≥ 12), APACHE II (< 28 vs ≥ 28) and testing for interactions using multiplicative terms. We used restricted cubic spline (RCS) models to explore potential non-linear associations. All analyses were performed using R software (version 3.3.2; R Foundation for Statistical Computing, Vienna, Austria) and Free Statistics software version 2.1.1. A two-tailed *p*-value <0.05 was considered statistically significant.

## 3 Results

### 3.1 Baseline characteristics of the study population

Table 1 summarizes the baseline characteristics of the study population. A total of 773 patients with S-AKI were included and stratified by GNRI tertiles. The mean age was 63.6 years, and 61.8% were male. Nutritional parameters showed clear gradients across tertiles. Albumin (2.1 ± 0.4 vs. 3.1 ± 0.5 g/dL), hemoglobin (9.3 ± 2.0 vs. 10.0 ± 2.3 g/dL), and BMI (20.7 ± 3.0 vs. 26.8 ± 4.2 kg/m^2^) all increased with higher GNRI (all *p* < 0.001). Lower GNRI was also associated with greater disease severity (APACHE II: 28.4 vs. 26.1, *p* = 0.003) and higher mortality (28-day: 71.5% vs. 50.4%; 90-day: 81.6% vs. 63.2%, both *p* < 0.001). CRP did not differ significantly among groups, but patients in the lowest GNRI tertile tended to have higher CRP levels, suggesting a greater inflammatory burden. Overall, GNRI was associated with both nutritional status and clinical outcomes in patients with S-AKI. Additionally, the detailed results of the univariate analysis can be found in Supplementary Table 1.

**TABLE 1:** Baseline characteristics of the study participants, overall and stratified by tertiles of the Geriatric Nutritional Risk Index (GNRI) score.

Variables	Total	Tertiles of GNRI*			*P*-Value
		<77.3	77.0–87.2	≥87.2	
Number	773	256	259	258	
**Demographic data**				
Age (years), mean ± SD	63.6 ± 14.1	64.7 ± 13.9	64.1 ± 13.6	62.1 ± 14.7	0.086
Male, *n* (%)	478 (61.8)	164 (64.1)	165 (63.7)	149 (57.8)	0.253
BMI (kg/m^2^), mean ± SD	23.7 ± 4.2	20.7 ± 3.0	23.5 ± 2.8	26.8 ± 4.2	<0.001
MAP (mmHg), mean ± SD	77.5 ± 14.5	77.4 ± 15.2	78.3 ± 14.4	76.9 ± 13.8	0.512
**Comorbidities**				
MI, *n* (%)	66 (8.5)	20 (7.8)	18 (6.9)	28 (10.9)	0.249
CHF, *n* (%)	140 (18.1)	40 (15.6)	46 (17.8)	54 (20.9)	0.291
PVD, *n* (%)	30 (3.9)	12 (4.7)	12 (4.6)	6 (2.3)	0.285
Dementia, *n* (%)	26 (3.4)	13 (5.1)	11 (4.2)	2 (0.8)	0.016
DM, *n* (%)	262 (33.9)	84 (32.8)	89 (34.4)	89 (34.5)	0.904
HTN, *n* (%)	412 (53.3)	143 (55.9)	136 (52.5)	133 (51.6)	0.590
COPD, *n* (%)	68 (8.8)	26 (10.2)	24 (9.3)	18 (7)	0.422
CCI, median (IQR)	3.0 (2.0,5.0)	3.0 (2.0,5.0)	3.0 (2.0,4.0)	3.0 (2.0,4.0)	0.568
**Laboratory parameters**				
K (mmol/L), mean ± SD	4.7 ± 1.1	4.7 ± 1.1	4.6 ± 1.1	4.8 ± 1.1	0.200
HCO_3_ (mmol/L), mean ± SD	16.8 ± 5.5	16.6 ± 5.6	17.1 ± 5.5	16.6 ± 5.5	0.528
Phosphate (mg/dL), mean ± SD	5.6 ± 2.2	5.2 ± 2.0	5.8 ± 2.3	5.8 ± 2.3	0.005
MV_CRRT, *n* (%)	611 (79.0)	215 (84)	206 (79.5)	190 (73.6)	0.015
Hemoglobin (g/dL), mean ± SD	9.7 ± 2.2	9.3 ± 2.0	9.7 ± 2.1	10.0 ± 2.3	0.001
BUN (mg/dL), mean ± SD	57.1 ± 29.2	57.8 ± 28.5	59.6 ± 31.6	53.9 ± 27.0	0.078
Cr (mg/dL), mean ± SD	2.7 ± 1.5	2.4 ± 1.3	2.8 ± 1.4	2.8 ± 1.6	0.008
ALB (g/dL), mean ± SD	2.6 ± 0.6	2.1 ± 0.4	2.6 ± 0.3	3.1 ± 0.5	<0.001
CRP (mg/dL), mean ± SD	70.3 (19.2,167.2)	80.9 (24.7,175.0)	72.2 (17.7,168.3)	55.0 (17.3,151.0)	0.103
eGFR, median (IQR)	26.3 (17.2,38.9)	29.4 (19.4,42.9)	24.8 (15.8,36.2)	25.9 (16.1,38.4)	0.002
UO_2 h (mL), mean ± SD	30.0 (5.0,100.0)	25.0 (4.8,86.2)	35.0 (5.0,100.0)	35.0 (10.0,100.0)	0.067
GNRI, median (IQR)	83.0 ± 12.0	70.6 ± 6.0	82.2 ± 2.8	96.1 ± 8.1	<0.001
**Disease severity score**				
APACHE II, median (IQR)	27.3 ± 8.0	28.4 ± 7.3	27.5 ± 8.0	26.1 ± 8.4	0.003
SOFA, median (IQR)	12.0 ± 3.6	12.0 ± 3.7	12.4 ± 3.2	11.6 ± 3.7	0.046
CRRT dose (mL/kg), mean ± SD	36.7 ± 4.8	37.4 ± 4.6	36.7 ± 4.9	36.1 ± 4.7	0.010
AKIN, *n* (%)					0.891
Stage 2	209 (27.0)	71 (27.7)	71 (27.4)	67 (26)	
Stage 3	564 (73.0)	185 (72.3)	188 (72.6)	191 (74)	
Status 28 day, *n* (%)	477 (61.7)	183 (71.5)	164 (63.3)	130 (50.4)	<0.001
Status 90 day, *n* (%)	564 (73.0)	209 (81.6)	192 (74.1)	163 (63.2)	<0.001

GNRI, Geriatric Nutritional Risk Index; BMI, body mass index; MAP, mean arterial pressure; MI, myocardial infarction; CHF, congestive heart failure; PVD, peripheral vascular disease; DM, diabetes mellitus; HTN, hypertension; COPD, chronic obstructive pulmonary disease; CCI, Charlson Comorbidity Index; K, potassium; HCO3, bicarbonate; MV, mechanical ventilation; Hb, hemoglobin; BUN, blood urea nitrogen; Cr, creatinine; ALB, albumin; CRP, C-reactive protein; eGFR, estimated glomerular filtration rate; UO_2 h, urine output within 2 h; APACHE II, Acute Physiology and Chronic Health Evaluation II; SOFA, Sequential Organ Failure Assessment; CRRT, continuous renal replacement therapy; AKIN, Acute Kidney Injury Network.

*Kruskal-Wallis H test was used to compare three categories of GNRI.

### 3.2 Kaplan–Meier survival analysis

**FIGURE 2:**
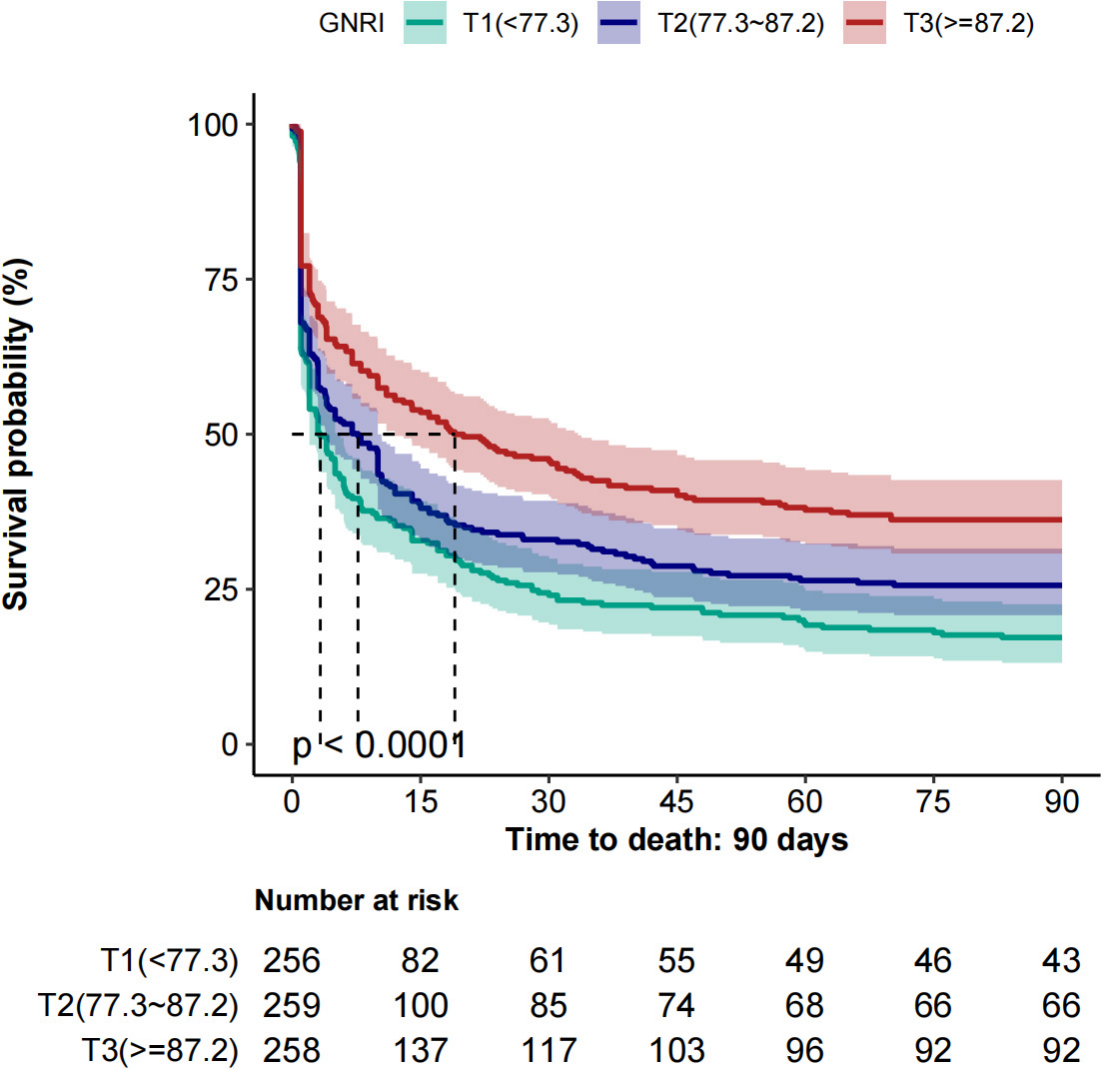
Kaplan–Meier survival curves for 28-day and 90-day all-cause mortality across GNRI tertiles. GNRI, Geriatric Nutritional Risk Index.

As shown in Figure 2, Kaplan–Meier survival curves illustrate 28-day and 90-day all-cause mortality across GNRI tertiles. Survival probability decreased progressively from tertile3 to tertile1, with patients in tertile1 (GNRI < 77.3) showing significantly reduced survival compared with those in tertile2 and tertile3. Log-rank tests confirmed significant differences at both 28 and 90 days (*p* < 0.001 for both).

### 3.3 Association between GNRI and short-term mortality

Table 2 shows the association between GNRI and all-cause mortality at 28 and 90 days. As a continuous variable, each one-unit increase in GNRI was associated with lower 28-day mortality (HR = 0.98, 95% CI: 0.97–0.99, *p* < 0.001). When categorized by tertiles, patients in tertile 3 (GNRI ≥ 87.2) had significantly lower mortality compared with tertile 1 (GNRI < 77.3). In the fully adjusted model, hazard ratios for 28-day and 90-day mortality in tertile 3 were 0.53 (95% CI: 0.42–0.67, *p* < 0.001) and 0.50 (95% CI: 0.41–0.63, *p* < 0.001), respectively. Trend analysis confirmed significant associations across tertiles (*p* for trend < 0.001). The proportional hazards assumption of the Cox models was tested and satisfied, as indicated by Schoenfeld residuals (all *p* > 0.05; Supplementary Figure 1). Restricted cubic spline (RCS) analyses were further performed to assess the dose–response relationship between GNRI and mortality (Supplementary Figure 2).

**TABLE 2:** Multivariable COX regression analysis of 28-day and 90-day mortality.

Exposure	28-dall-cause death				90-dall-cause death			
	Crude	Model I	Model II	Model III	Crude	Model I	Model II	Model III
GNRI	0.98 (0.97∼0.99)	0.98 (0.97∼0.99)	0.98 (0.97∼0.99)	0.98 (0.97∼0.98)	0.98 (0.97∼0.99)	0.98 (0.97∼0.98)	0.98 (0.97∼0.98)	0.97 (0.97∼0.98)
*P*	<0.001	<0.001	<0.001	<0.001	<0.001	<0.001	<0.001	<0.001
**GNRI tertiles**								
Tertile 1	1 (Ref)	1 (Ref)	1 (Ref)	1 (Ref)	1 (Ref)	1 (Ref)	1 (Ref)	1 (Ref)
Tertile 2	0.8 (0.64∼0.98)	0.8 (0.65∼0.99)	0.8 (0.65∼0.99)	0.69 (0.56∼0.86)	0.79 (0.65∼0.96)	0.78 (0.64∼0.95)	0.78 (0.64∼0.95)	0.69 (0.56∼0.84)
*P*	0.034	0.036	0.038	<0.001	0.018	0.015	0.013	<0.001
Tertile 3	0.59 (0.47∼0.74)	0.59 (0.47∼0.74)	0.58 (0.47∼0.73)	0.53 (0.42∼0.67)	0.57 (0.46∼0.7)	0.56 (0.46∼0.69)	0.54 (0.44∼0.67)	0.5 (0.41∼0.63)
*P*	<0.001	<0.001	<0.001	<0.001	<0.001	<0.001	<0.001	<0.001
*P* for trend	<0.001	<0.001	<0.001	<0.001	<0.001	<0.001	<0.001	<0.001

Crude: unadjusted model. Model I: adjusted for age and sex. Model II: Model I + MAP, MI, CHF, PVD, dementia, DM, HTN, and COPD. Model III: Mode II + phosphate, hemoglobin, CRP, APACHE II score, SOFA score, CRRT dose, and AKIN stage. GNRI, Geriatric Nutritional Risk Index; MAP, mean arterial pressure; Ml, myocardial infarction; CHF, congestive heart failure; PVD, peripheral vascular disease; DM, diabetes mellitus; HTN, hypertension; COPD, chronic obstructive pulmonary disease; CRP, C-reactive protein; APACHE II, Acute Physiology and Chronic Health Evaluation II; SOFA, Sequential Organ Failure Assessment score; CRRT, continuous renal replacement therapy; AKIN, Acute Kidney Injury Network.

### 3.4 Subgroup analysis

**FIGURE 3:**
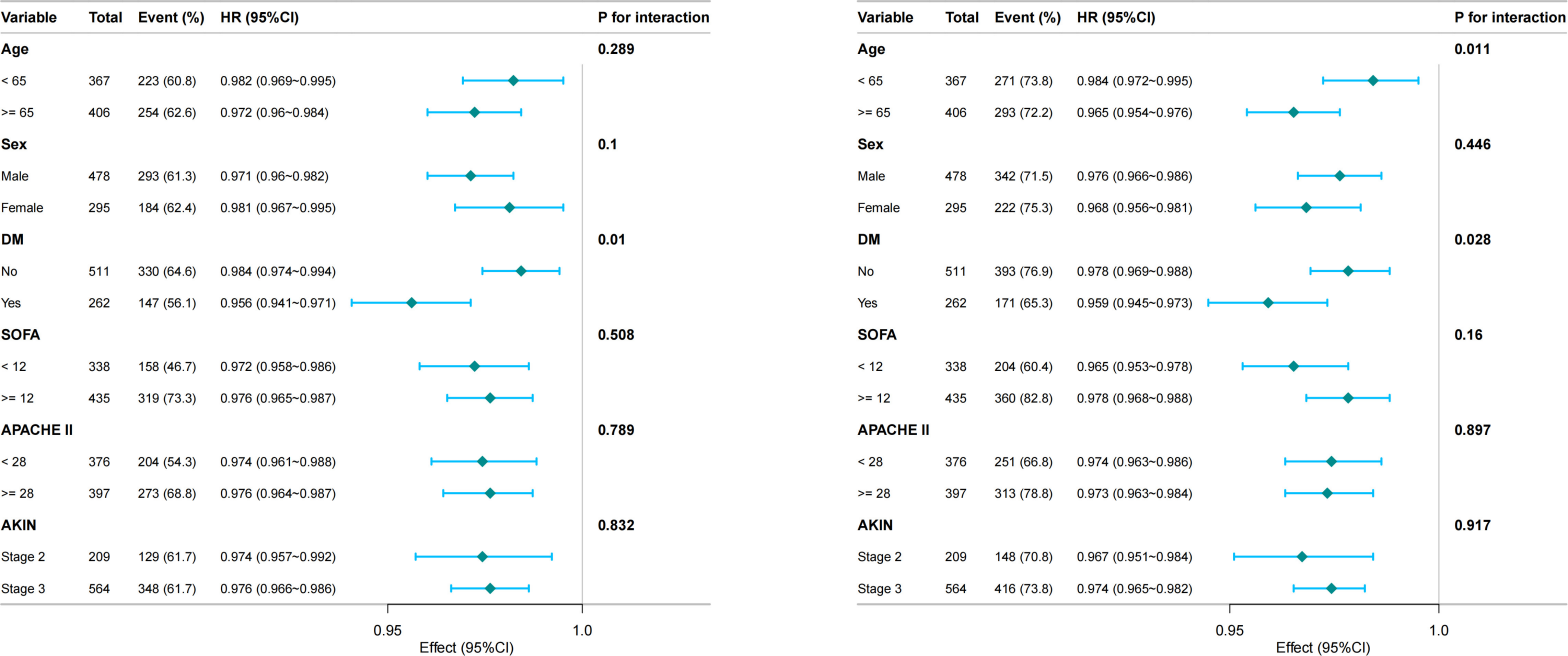
Subgroup analyses of the association between GNRI and 28-day (Left) and 90-day (Right) mortality. GNRI, Geriatric Nutritional Risk Index; DM, diabetes mellitus; SOFA, Sequential Organ Failure Assessment; APACHE II, Acute Physiology and Chronic Health Evaluation II; AKIN, acute kidney injury network.

To examine the robustness of the association between GNRI and short-term mortality, subgroup analyses were performed across a range of clinically relevant strata, including age, sex, diabetes mellitus, severity of illness score and AKIN stage (Figure 3). In most subgroups, the inverse relationship between GNRI and mortality remained consistent, indicating a stable predictive value across diverse patient profiles. No significant interactions were detected (all P interaction >0.05 for 28-day; except age, *P* = 0.028 for 90-day), indicating the robustness of the GNRI-mortality association across clinical subgroups.

### 3.5 ROC curve analysis and predictive performance of GNRI

**FIGURE 4:**
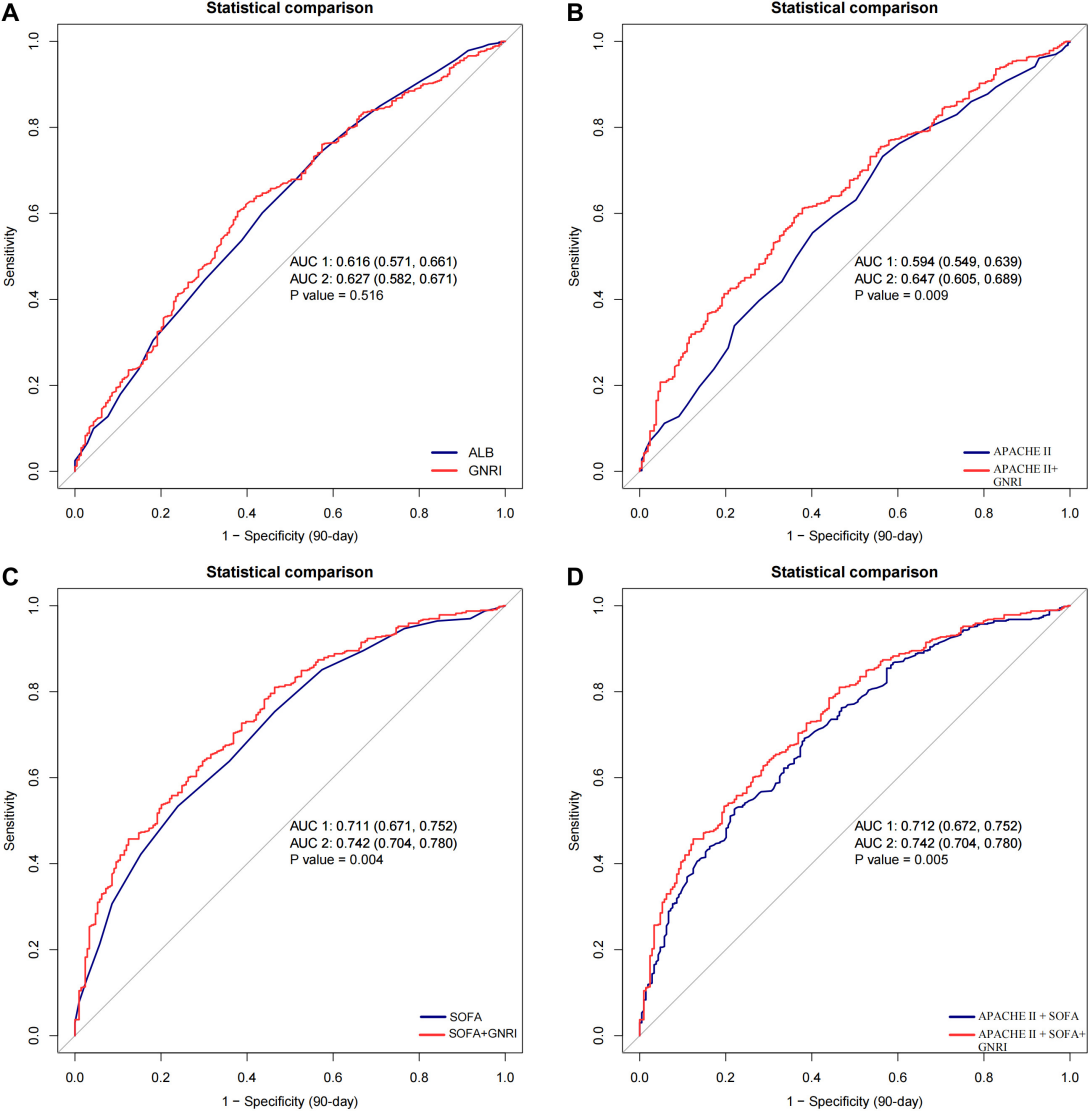
Receiver operating characteristic (ROC) curves for predicting 90-day mortality in patients with S-AKI receiving CRRT. **(A)** GNRI versus albumin; **(B)** APACHE II versus APACHE II + GNRI; **(C)** SOFA versus SOFA + GNRI; **(D)** APACHE II + SOFA versus APACHE II + SOFA + GNRI. The addition of GNRI to conventional severity scores improved predictive performance, as reflected by higher AUC values. GNRI, Geriatric Nutritional Risk Index; ALB, albumin; APACHE II, Acute Physiology and Chronic Health Evaluation II; SOFA, Sequential Organ Failure Assessment; CRRT, continuous renal replacement therapy; AUC, area under the curve.

The predictive performance of GNRI was further evaluated using ROC curve analysis. For 90-day mortality, GNRI achieved an AUC of 0.627 (95% CI: 0.582–0.671), higher than albumin (0.616) and APACHE II (0.594) but lower than SOFA (0.711). Incorporating GNRI into APACHE II improved the AUC to 0.647 (*p* = 0.009), accompanied by significant improvements in NRI (0.210, *p* < 0.001) and IDI (0.036, *p* < 0.001). Similarly, adding GNRI to SOFA increased the AUC from 0.711 to 0.742 (*p* = 0.004), with significant improvements in both NRI and IDI (all *p* < 0.001). The combined APACHE II + SOFA model also benefited from the inclusion of GNRI (AUC: 0.742 vs. 0.712, *p* = 0.005) (Figure 4 and Supplementary Table 2). Comparable findings were observed for 28-day mortality, as shown in Supplementary Figure 3 and Supplementary Table 3.

## 4 Discussion

In this study, we found that a lower GNRI was independently linked to higher short-term mortality in critically ill patients with S-AKI receiving CRRT. This association remained consistent across subgroups, especially in older adults and those with diabetes. However, GNRI should not be viewed as a marker of nutrition alone. Serum albumin, one of its main components, is also a negative acute-phase reactant and decreases in response to systemic inflammation. These results suggest that GNRI is associated with prognosis and may serve as a useful marker in this high-risk population, but causality cannot be inferred.

Our findings are consistent with previous studies that have demonstrated the prognostic value of the GNRI in various clinical settings. Ono et al. (21) studied GNRI in patients with acute decompensated heart failure. Lower GNRI at discharge predicted higher long-term mortality. Although their setting and timing differed, the direction of association aligns with our findings. More recently, several observational studies have reported similar associations between higher GNRI scores and reduced mortality in patients with acute kidney injury (AKI) (22). This trend is further supported by a study conducted by Liao et al. (23), who stratified elderly ICU patients with AKI using a GNRI cutoff value of 98. They found that patients in the higher GNRI group had significantly lower one-year mortality rates. Although the GNRI threshold used in their study differs from that in our analysis, the overall direction of the association remains consistent with our results. Cai et al. (24), analyzed 4,515 patients with S-AKI from the MIMIC-IV database. They found that higher GNRI was independently associated with lower 28-day mortality. Their cohort included all S-AKI patients, not just those treated with CRRT. Even so, the results are consistent with ours and reinforce the prognostic value of GNRI in this population.

GNRI incorporates serum albumin and body weight relative to ideal weight, both of which reflect nutritional and inflammatory status (8). Compared with traditional markers such as BMI or isolated albumin levels, GNRI provides a more integrated and stable estimate of nutritional reserve (25–27). This is particularly important in critically ill patients, where fluid overload and systemic inflammation can distort anthropometric or laboratory measurements (7). Previous studies have also shown that serum albumin is a reliable prognostic biomarker in AKI patients receiving CRRT (28). Moreover, Ruan et al. (29) demonstrated that GNRI predicted outcomes largely through its association with inflammatory severity in elderly patients with cancer cachexia Zhao et al. (30) found that lower GNRI values were associated with worse hospitalization outcomes after cardiac surgery, again highlighting the role of inflammation. Similarly, He et al. (31) showed that the lactate/albumin ratio, another marker integrating inflammatory and nutritional status, was independently associated with 28-day mortality in SA-AKI patients. More recently, other composite indices have also been reported in sepsis and AKI. The neutrophil-to-PNI ratio (32), glucose-to-potassium ratio (GPR) (33), and triglyceride-glucose (TyG) index (34) were all associated with higher mortality. Together with our results, these findings highlight the value of simple markers that integrate inflammation, metabolism, and nutrition for risk stratification in critically ill patients. In addition GNRI is simple to calculate at the bedside and does not rely on subjective assessment, making it suitable for routine use in the ICU. Given the high mortality rates in S-AKI despite advances in renal support therapies, incorporating GNRI into standard risk assessment protocols could enhance individualized care planning and resource allocation. This approach is in line with a growing emphasis on multidimensional prognostic models in critical care. For example, Hu et al. (35) conducted a prospective cohort of 1,736 elderly non-cardiac surgical patients. They found that combining cardiac biomarkers with the AUB-HAS2 risk score improved prediction of major adverse cardiovascular events. This highlights the broader importance of multidimensional prognostic models.

Beyond nephrology and critical care, simple biochemical and clinical indices have also been investigated in other medical fields. In reproductive endocrinology, indicators such as the progesterone to mature oocyte index (PMOI), anti-Müllerian hormone (AMH), antral follicle count (AFC) and even blood type have been examined as predictors of ovarian response and pregnancy outcomes in women undergoing IVF/ICSI (36–39). These studies demonstrate that such markers can serve as independent prognostic tools. In oncology, nutritional indices such as the prognostic nutritional index (PNI) and GNRI have been validated as predictors of survival in lung cancer patients receiving immunotherapy and in esophageal cancer patients treated with chemoradiotherapy (40, 41). Although these investigations were conducted in distinct patient populations, they consistently highlight the predictive value of simple and cost-effective indices. Collectively, these cross-disciplinary findings reinforce the broader applicability of GNRI as a practical prognostic marker and support its potential role in guiding clinical decisions for patients with S-AKI receiving CRRT.

Nevertheless, this study has several limitations. First, the analysis was retrospective and based on secondary data from a single public database. This design restricts control over data quality and unmeasured confounding. Second, the cohort included only patients with severe S-AKI treated with CRRT. Therefore, the results cannot be extended to milder forms of AKI or to patients managed without dialysis. Third, the study did not assess other important outcomes, including renal recovery, ICU length of stay, long-term survival, or repeated admissions. In addition, multiple subgroup analyses were performed, which may increase the risk of type I error. These limitations suggest that the findings should be interpreted with caution. Future prospective studies are needed to validate our findings and to test whether GNRI-guided interventions can improve outcomes.

## 5 Conclusion

In conclusion, GNRI independently predicts 28- and 90-day all-cause mortality in S-AKI patients receiving CRRT. Lower GNRI scores are associated with significantly higher short-term mortality. Further prospective studies are needed to validate its prognostic utility and to explore the biological mechanisms underlying its association with outcomes in S-AKI.

## Data Availability

The original contributions presented in this study are included in this article/Supplementary material, further inquiries can be directed to the corresponding authors.
