# Supplementary material for: Prognostic value of the Geriatric Nutritional Risk Index in sepsis-associated acute kidney injury: a retrospective cohort study
Source: Front Nutr. 2025 Nov 21;12:1635568. doi: 10.3389/fnut.2025.1635568 (PMC12678099; doi:10.3389/fnut.2025.1635568)
Supplement: Supplementary file 1 [file Data_Sheet_1.zip › Supplementary_Figures_Tables/Table S1. Univariate regression analysis.docx]

Table S1. Univariate regression analysis(28-day and 90-day all-cause mortality)

| Variables | 28-day mortality | | 90-day mortality | |
| --- | --- | --- | --- | --- |
|  | HR(95%CI) | P value | HR(95%CI) | P value |
| Age(years) (cont. var.) | 1.0032 (0.9969,1.0095) | 0.32 | 1.0033 (0.9975,1.0092) | 0.263 |
| Sex: Female vs Male | 1.01 (0.84,1.22) | 0.895 | 1.11 (0.93,1.31) | 0.239 |
| BMI(kg/m^2^) (cont. var.) | 0.98 (0.96,1) | 0.052 | 0.97 (0.95,0.99) | 0.003 |
| MAP(mmHg) (cont. var.) | 0.99 (0.98,0.99) | < 0.001 | 0.99 (0.98,0.99) | < 0.001 |
| MI: Yes vs No | 0.74 (0.52,1.04) | 0.083 | 0.78 (0.57,1.07) | 0.119 |
| CHF: Yes vs No | 0.78 (0.61,1) | 0.046 | 0.88 (0.7,1.09) | 0.238 |
| PVD: Yes vs No | 0.87 (0.54,1.4) | 0.569 | 1.02 (0.66,1.58) | 0.915 |
| Dementia: Yes vs No | 0.68 (0.39,1.17) | 0.165 | 0.7 (0.42,1.17) | 0.173 |
| DM: Yes vs No | 0.78 (0.64,0.94) | 0.011 | 0.74 (0.62,0.89) | 0.001 |
| HTN: Yes vs No | 0.72 (0.6,0.87) | < 0.001 | 0.75 (0.64,0.89) | < 0.001 |
| COPD: Yes vs No | 0.71 (0.5,1.01) | 0.055 | 0.75 (0.55,1.02) | 0.07 |
| CCI (cont. var.) | 1.08 (1.04,1.12) | < 0.001 | 1.06 (1.02,1.09) | 0.003 |
| K(mmol/L) (cont. var.) | 0.9973 (0.9151,1.0868) | 0.95 | 1.03 (0.95,1.12) | 0.423 |
| HCO_3_(mmol/L) (cont. var.) | 0.99 (0.97,1) | 0.161 | 0.9928 (0.9775,1.0084) | 0.364 |
| Phosphate(mg/dL) (cont. var.) | 1.09 (1.05,1.13) | < 0.001 | 1.07 (1.04,1.11) | < 0.001 |
| MV_CRRT: Yes vs No | 1.71 (1.33,2.18) | < 0.001 | 1.5 (1.21,1.87) | < 0.001 |
| Hemoglobin(g/dL) (cont. var.) | 0.95 (0.91,0.99) | 0.027 | 0.96 (0.92,0.99) | 0.026 |
| BUN(mg/dL) (cont. var.) | 1.0022 (0.9994,1.0051) | 0.128 | 1.0022 (0.9996,1.0048) | 0.097 |
| Cr(mg/dL) (cont. var.) | 0.9 (0.85,0.96) | 0.002 | 0.89 (0.83,0.94) | < 0.001 |
| ALB(g/dL) (cont. var.) | 0.65 (0.55,0.76) | < 0.001 | 0.63 (0.54,0.74) | < 0.001 |
| CRP(mg/dL) (cont. var.) | 1.0009 (1.0001,1.0017) | 0.031 | 1.0005 (0.9998,1.0013) | 0.187 |
| eGFR (cont. var.) | 1.002 (0.9984,1.0056) | 0.283 | 1.0039 (1.0008,1.007) | 0.015 |
| UO_2hrs(mL) (cont. var.) | 0.9966 (0.9954,0.9978) | < 0.001 | 0.9974 (0.9964,0.9985) | < 0.001 |
| GNRI (cont. var.) | 0.98 (0.97,0.99) | < 0.001 | 0.98 (0.97,0.99) | < 0.001 |
| APACHE II (cont. var.) | 1.03 (1.02,1.04) | < 0.001 | 1.02 (1.01,1.03) | < 0.001 |
| SOFA (cont. var.) | 1.16 (1.13,1.2) | < 0.001 | 1.11 (1.08,1.14) | < 0.001 |
| CRRT dose(mL/kg) (cont. var.) | 1.0037 (0.9841,1.0236) | 0.716 | 0.9984 (0.9813,1.0157) | 0.853 |
| AKIN: Stage3 vs Stage2 | 1.03 (0.84,1.26) | 0.788 | 1.0092 (0.8365,1.2175) | 0.924 |

Notes:BMI, body mass index; MAP, mean arterial pressure; Ml, myocardial infarction; CHF, congestive heart failure; PVD, peripheral vascular disease; DM, diabetes mellitus; HTN, hypertension; COPD, chronic obstructive pulmonary disease; CCl, Charlson Comorbidity index; HCO_3_, bicarbonate; MV, mechanical ventilation; BUN, blood urea nitrogen; Cr, creatinine level; ALB, albumin; CRP, C-reactive protein; eGFR, estimated glomerular filtration rate; UO_2hrs, 2h urine output; GNRI, Geriatric Nutritional Risk Index; APACHE II, Acute Physiology and Chronic Health Evaluation II; SOFA, Sequential Organ Failure Assessment ; CRRT, continuous renal replacement therapy; AKIN, Acute Kidney Injury Network.
