# Supplementary material for: Prognostic value of the Geriatric Nutritional Risk Index in sepsis-associated acute kidney injury: a retrospective cohort study
Source: Front Nutr. 2025 Nov 21;12:1635568. doi: 10.3389/fnut.2025.1635568 (PMC12678099; doi:10.3389/fnut.2025.1635568)
Supplement: Supplementary file 1 [file Data_Sheet_1.zip › Supplementary_Figures_Tables/Table S2. Comparison of the AUC and predictive power of prognosis for 90-day mortality.docx]

Table S2. Distribution of Mortality Outcomes Across CAR Tertiles in S-AKI Patients Receiving CRRT.

| Variables | AUC (95% CI) | p value | NRI (95% CI) | p value | IDI (95% CI) | p value |
| --- | --- | --- | --- | --- | --- | --- |
| ALB | 0.616(0.571~0.661) | Reference | Reference |  | Reference |  |
| GNRI | 0.627(0.582~0.671) | 0.516 | 0.006(-0.115~0.133) | 0.85 | 0.002(-0.017~0.022) | 0.802 |
| APACHE II | 0.594(0.549~0.639) | Reference | Reference |  | Reference |  |
| APACHE II + GNRI | 0.647(0.605~0.689) | 0.009 | 0.21(0.13~0.284) | 0.0 | 0.036(0.017~0.066) | 0.0 |
| SOFA | 0.711(0.671~0.752) | Reference | Reference |  | Reference |  |
| SOFA + GNRI | 0.742(0.704~0.780) | 0.004 | 0.215(0.131~0.292) | 0.0 | 0.036(0.016~0.062) | 0.0 |
| APACHE II + SOFA | 0.712(0.672~0.752) | Reference | Reference |  | Reference |  |
| APACHE II + SOFA + GNRI | 0.742(0.704~0.780) | 0.005 | 0.211(0.134~0.287) | 0.0 | 0.035(0.016~0.06) | 0.0 |

Abbreviations: AUC, area under the curve; CI, confidence interval; NRI, net reclassification improvement; IDI, integrated discrimination improvement; ALB, albumin; GNRI, Geriatric Nutritional Risk Index; APACHE II, Acute Physiology and Chronic Health Evaluation II; SOFA, Sequential Organ Failure

Assessment.
