# Supplementary material for: Prognostic value of the Geriatric Nutritional Risk Index in sepsis-associated acute kidney injury: a retrospective cohort study
Source: Front Nutr. 2025 Nov 21;12:1635568. doi: 10.3389/fnut.2025.1635568 (PMC12678099; doi:10.3389/fnut.2025.1635568)
Supplement: Supplementary file 1 [file Data_Sheet_1.zip › Supplementary_Figures_Tables/Table S3. Comparison of the AUC and predictive power of prognosis for 28-day mortality.docx]

Table S3. Distribution of Mortality Outcomes Across CAR Tertiles in S-AKI Patients Receiving CRRT.

| Variables | AUC (95% CI) | p value | NRI (95% CI) | p value | IDI (95% CI) | p value |
| --- | --- | --- | --- | --- | --- | --- |
| ALB | 0.610(0.569~0.651) | Reference | Reference |  | Reference |  |
| GNRI | 0.613(0.572~0.653) | 0.853 | 0.001(-0.08~-0.079) | 1.026 | -0.002(-0.015~0.012) | 0.699 |
| APACHE II | 0.596(0.556~0.637) | Reference | Reference |  | Reference |  |
| APACHE II + GNRI | 0.642(0.603~0.682) | 0.006 | 0.086(-0.001~0.137) | 0.056 | 0.013(0.001~0.032) | 0.02 |
| SOFA | 0.699(0.661~0.737) | Reference | Reference |  | Reference |  |
| SOFA + GNRI | 0.726(0.690~0.762) | 0.004 | 0.091(-0.001~0.149) | 0.056 | 0.013(-0.0~0.035) | 0.056 |
| APACHE II + SOFA | 0.701(0.663~0.739) | Reference | Reference |  | Reference |  |
| APACHE II + SOFA + GNRI | 0.726(0.690~0.762) | 0.006 | 0.087(0.003~0.148) | 0.04 | 0.012(-0.0~0.033) | 0.068 |

Abbreviations: AUC, area under the curve; CI, confidence interval; NRI, net reclassification improvement; IDI, integrated discrimination improvement; ALB, albumin; GNRI, Geriatric Nutritional Risk Index; APACHE II, Acute Physiology and Chronic Health Evaluation II; SOFA, Sequential Organ Failure

Assessment.
