# Supplementary figures and images for: Prognostic value of the Geriatric Nutritional Risk Index in sepsis-associated acute kidney injury: a retrospective cohort study
Source: Front Nutr. 2025 Nov 21;12:1635568. doi: 10.3389/fnut.2025.1635568 (PMC12678099; doi:10.3389/fnut.2025.1635568)

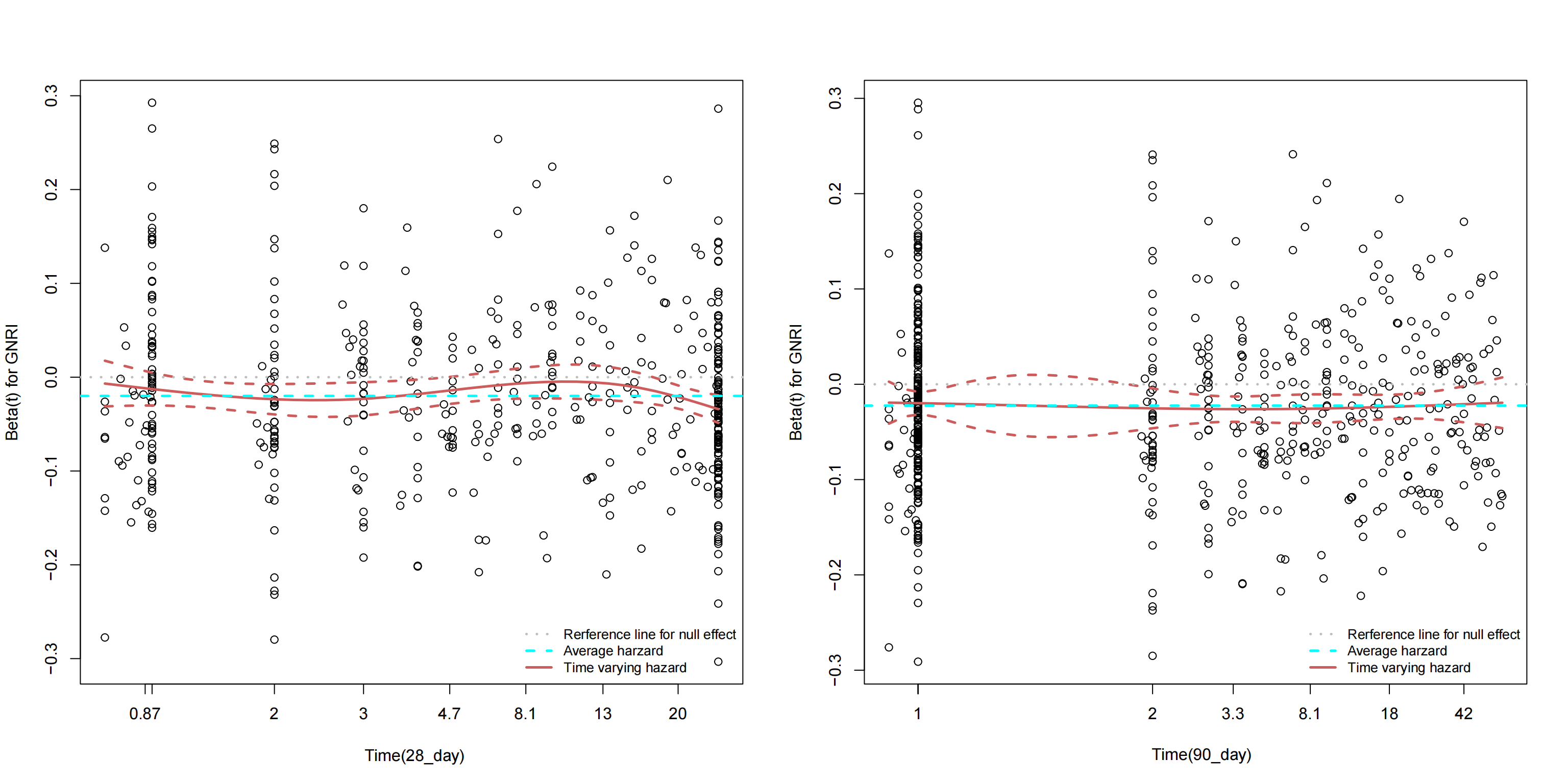

Supplement: Supplementary file 1 [file Data_Sheet_1.zip › Supplementary_Figures_Tables/Figure S1. Proportional Hazards Assumption for GNRI (28- & 90-Day Mortality).tiff]

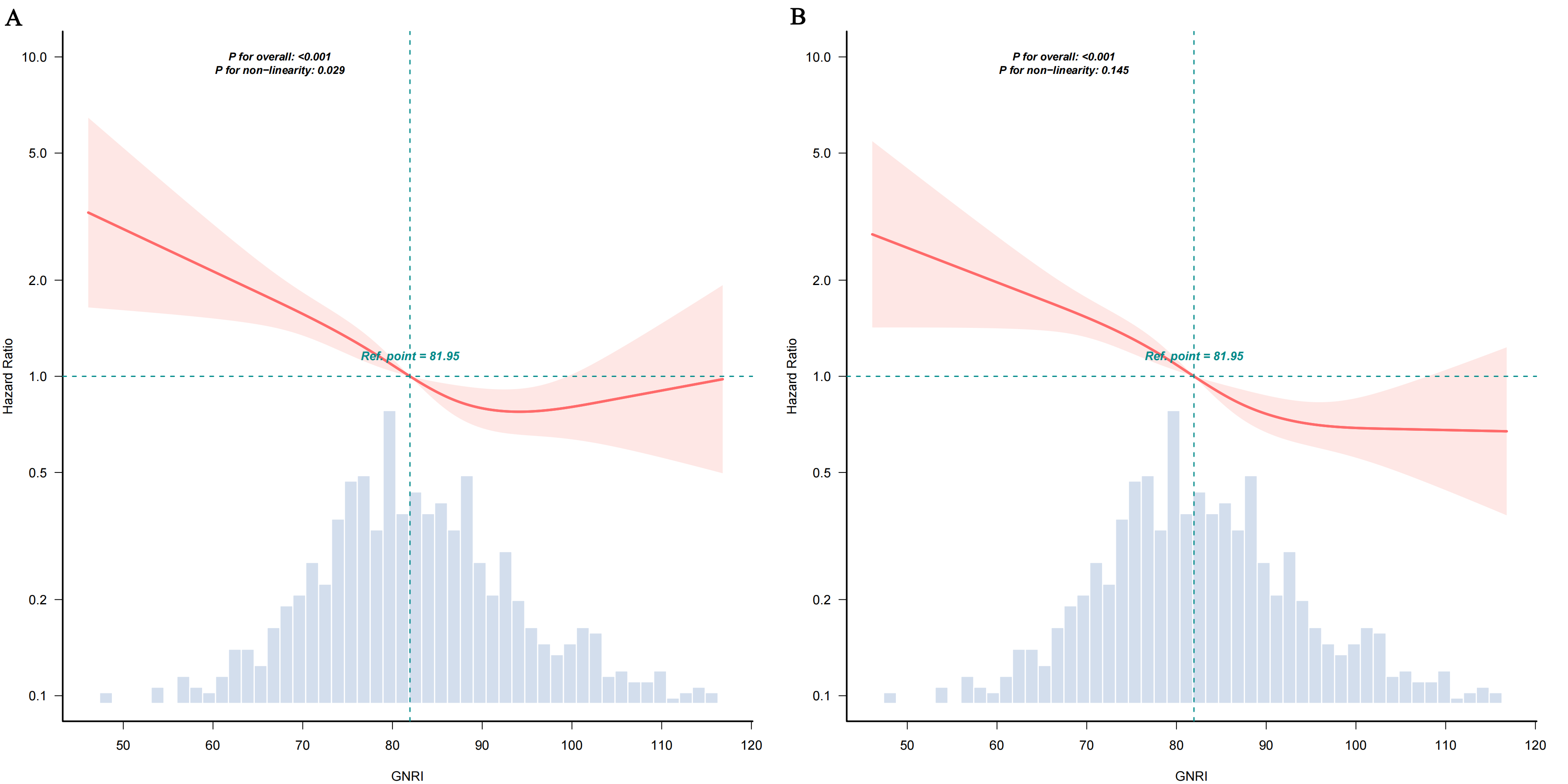

Supplement: Supplementary file 1 [file Data_Sheet_1.zip › Supplementary_Figures_Tables/Figure S2. 28- &90-day fitting curves.tiff]

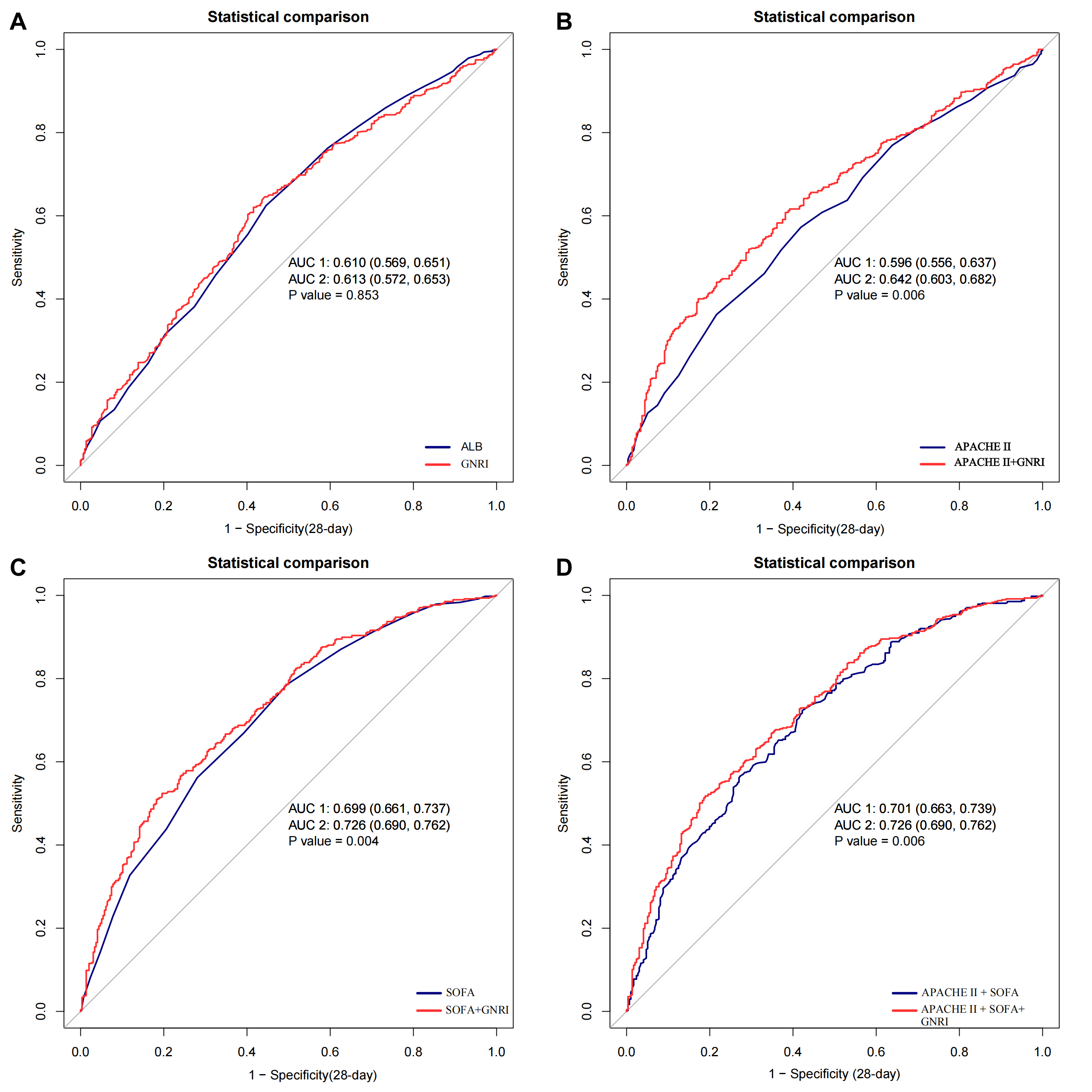

Supplement: Supplementary file 1 [file Data_Sheet_1.zip › Supplementary_Figures_Tables/Figure S3. ROC curves for predicting 28-day mortality.tiff]
